# *In Situ* Bioprinting of Autologous Skin Cells Accelerates Wound Healing of Extensive Excisional Full-Thickness Wounds

**DOI:** 10.1038/s41598-018-38366-w

**Published:** 2019-02-12

**Authors:** Mohammed Albanna, Kyle W. Binder, Sean V. Murphy, Jaehyun Kim, Shadi A. Qasem, Weixin Zhao, Josh Tan, Idris B. El-Amin, Dennis D. Dice, Julie Marco, Jason Green, Tao Xu, Aleksander Skardal, James H. Holmes, John D. Jackson, Anthony Atala, James J. Yoo

**Affiliations:** 1 Wake Forest Institute for Regenerative Medicine, Wake Forest School of Medicine, Medical Center Boulevard, Winston-Salem, NC 27157 USA; 2 Department of Pathology, Wake Forest School of Medicine, Medical Center Boulevard, Winston-Salem, NC 27157 USA; 3 Jiangsu Key Laboratory of Neuroregeneration, Nantong University, Nantong, China; 4 Department of Surgery, Wake Forest School of Medicine, Medical Center Boulevard, Winston-Salem, NC 27157 USA; 5 Virginia Tech-Wake Forest School of Biomedical Engineering and Sciences, Wake Forest School of Medicine, Winston-Salem, NC 27157 USA

## Abstract

The early treatment and rapid closure of acute or chronic wounds is essential for normal healing and prevention of hypertrophic scarring. The use of split thickness autografts is often limited by the availability of a suitable area of healthy donor skin to harvest. Cellular and non-cellular biological skin-equivalents are commonly used as an alternative treatment option for these patients, however these treatments usually involve multiple surgical procedures and associated with high costs of production and repeated wound treatment. Here we describe a novel design and a proof-of-concept validation of a mobile skin bioprinting system that provides rapid on-site management of extensive wounds. Integrated imaging technology facilitated the precise delivery of either autologous or allogeneic dermal fibroblasts and epidermal keratinocytes directly into an injured area, replicating the layered skin structure. Excisional wounds bioprinted with layered autologous dermal fibroblasts and epidermal keratinocytes in a hydrogel carrier showed rapid wound closure, reduced contraction and accelerated re-epithelialization. These regenerated tissues had a dermal structure and composition similar to healthy skin, with extensive collagen deposition arranged in large, organized fibers, extensive mature vascular formation and proliferating keratinocytes.

## Introduction

Chronic wounds such as diabetic, venous and pressure ulcers and burn wounds represent a burden to patients and surgeons, affecting over 7 million patients in the United States with an annual treatment expenditure of $25 billion. Chronic, large or non-healing wounds are especially costly because they often require multiple treatments; for example, a single diabetic foot ulcer can cost approximately $50,000 to treat^1^. Full thickness skin injuries are a major source of mortality and morbidity for civilians, with an estimated 500,000 civilian burns treated in the United States each year^2,3^. For military personnel, burn injuries account for 10–30% of combat casualties in conventional warfare^4^.

The early excision and the proper coverage of wounds are vital steps in increasing the survivability of patients with extensive burn injuries. Patients who suffer from these types of wounds respond best when rapid treatments are available that result in closure and protection of the wounds as fast as possible. Early treatment of wounds is crucial to prevent wounds from worsening with time and causing further tissue damage and long-term hypertrophic scarring. Patients who receive delayed treatments, or under-performing treatments, often are subject to extensive scarring that can result in long-term physiological defects such as disfigurements and loss of range of motion. Split thickness autografts are regarded as the “gold standard” technique for treating severe wounds^5^. However, the adequate coverage of wounds is often a challenge particularly when there is limited availability of healthy donor skin to harvest. Allografts are an option, but risk immune rejection of the graft^6^.

These limitations have led to the development of dermal substitutes, which are most often comprised of a synthetic or biological scaffold with, or without the inclusion of cells. Although such materials result in improved wound healing^7–9^ they are costly to produce and result in relatively poor cosmetic outcomes. Tissue engineering approaches have led to more complex biological skin equivalents as an alternative option to autografts^10–12^. The inclusion of both major skin cell types (keratinocytes and fibroblasts) in a single graft has been shown to improve skin regeneration in burn wounds^13,14^ and promote closure of chronic diabetic foot ulcers, when compared with standard therapies^15,16^. Unfortunately, these skin substitutes are also hard to produce with custom sizes and dimensions so lack the ability to adequately cover wounds with varying depth or topography.

Cellular therapy is a promising alternative to biological skin-equivalents. A successful cell-based technique could rapidly cover wounds and accelerate healing using living components. Epidermal keratinocytes and dermal fibroblasts can be easily isolated from a small biopsy of uninjured skin tissue^17^ and applied to the wound using a manually seeded matrix or with cell spraying methods^18–20^. Rapid wound coverage has been achieved through transplantation of a suspension of either freshly harvested or culture expanded keratinocytes at the time of wound debridement rather than the use of a coherent sheet of cells^21–23^. Additionally, it has been shown that delivery of cells to the wound using techniques such as cell spraying results in faster healing and better cosmetic outcomes than those repaired with non-cellular substitutes^24,25^. Unfortunately, the low delivery precision of current seeding and spraying technologies prevents the accurate delivery of specific cell types to the required target sites. As a result, these current techniques cannot generate the complex skin structure that would be required to obtain functional and aesthetically acceptable results.

In contrast to manual cell seeding or cell spraying, bioprinting has the capability to deliver cells to specific target sites using layer-by-layer freeform fabrication, and it has been applied in numerous applications^26^. Inkjet printers (also known as drop-on-demand printing) are the most commonly used type of printers for both non-biological and biological applications. Controlled volumes of liquid are delivered to pre-defined locations using use thermal^27,28^ or acoustic^29^ forces to eject drops of liquid from the cartridge onto a substrate. Cartridges can deliver a wide variety of biological materials, including cells, with high viability. In this study, we describe the design and a proof-of-concept validation of a novel mobile skin bioprinting system. The system integrates imaging technology to determine the topography of a wound with precise *in-situ* delivery of cells to tailor the technology to individual patient’s needs. Our device prints skin cells directly into an injured wound, delivering dermal fibroblasts and epidermal keratinocytes to specific locations of the wound, replicating the layered skin structure, and accelerating the formation of normal skin structure and function.

## Results

### Skin Bioprinter Design and Construction

**Figure 1:**
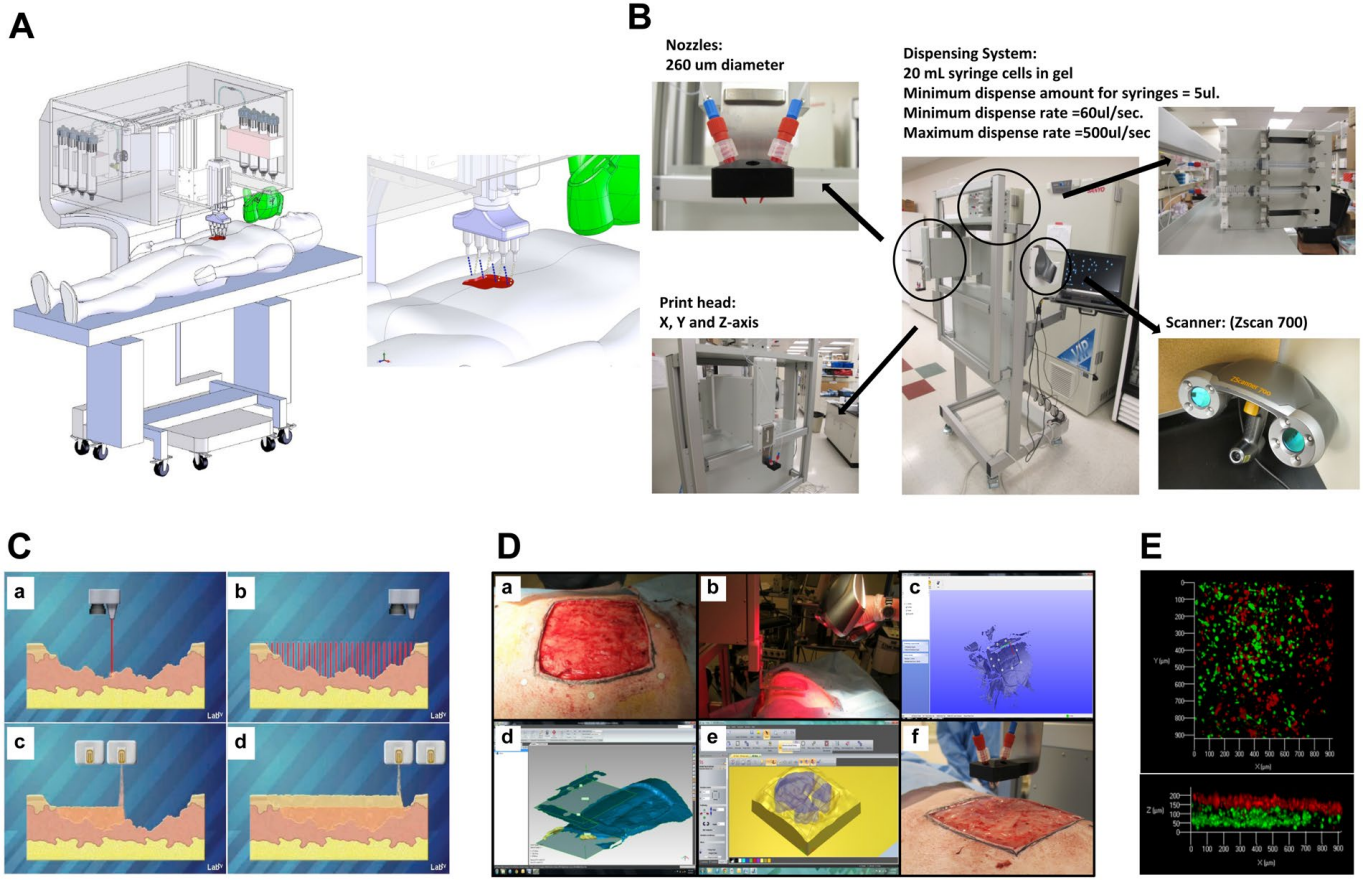
Skin bioprinter prototype and *in situ* bioprinting concept. (**A**) Schematic demonstrating scale, design and components of the skin bioprinter. (**B**) The main components of the system consist of 260 µm diameter nozzles, driven by up to 8 independently dispensing systems connected to a print-head with an XYZ movement system, in addition to the 3D wound scanner. All components are mounted on a frame small enough to be mobile in the operating room. (**C**) Skin bioprinting concept. Wounds are first scanned to obtain precise information on wound topography, which then guides the print-heads to deposit specified materials and cell types in appropriate locations (Images courtesy of LabTV - National Defense Education Program, Washington, D.C.). (**D**) Example of skin bioprinting process, where markers that are placed around the wound area used as reference points (a) prior to scanning with a hand-held ZScanner™ Z700 scanner (b). Geometric information obtained via scanning is then inputted in the form of an STL file to orient the scanned images to standard coordinate system (c). The scanned data with its coordinate system is used to generate the fill volume and the path points for nozzle head to travel to print the fill volume (d). Output code is then provided to the custom bioprinter control interface for generation of nozzle path needed to print fill volume (e,f). (**E**) This system facilitates the depositing of multiple cell types with high precision and control. Layering of fibroblasts (green) and keratinocytes (red) is shown.

Tissue engineering has had initial successes with building a number of tissues clinically, however, challenges still exist in developing and translating complex tissue systems that require coordinated and systemic function. Based on the shortcomings of the current technologies and therapies of wound treatments, we established design criteria for an *in situ* skin bioprinting system. The bioprinting system is: (1) portable and capable of being quickly transported, (2) able to identify and measure a wide range of wound sizes and topologies accurately, (3) capable of delivering multiple cell types to precise spatial orientation tailored to a individual wound, (4) easily sterilized, and (5) is easily operated and maintained with relatively low cost. A schematic demonstrating scale, design and components of the bioprinter system is shown in Fig. 1A. The main components of the system consist of a hand-held 3D wound scanner, and a print-head with an XYZ movement system containing eight 260 µm diameter nozzles, each driven by an independent dispensing motor. All components are mounted on a frame small enough to be mobile in the operating room (Fig. 1B). The dimensions of the system are 79 cm wide (patient head to toe direction) by 77 cm deep (cross patient direction). When the robotic arm is fully extended, it hands out over the base an additional 50 cm so the full reach is 127 cm, which is large enough to cover the torso of an average patient but small enough to easily pass through most doorframes. The skin bioprinter system is equipped with locks that engage with the base of the patient table to prevent movement while printing cells. Once the system is in position over the patient these locks restrain the mobility of the system during the printing process to ensure high delivery precision. The XYZ axes contribute to delivery precision by using a plotting system capable of 100 µm movements. In addition, the Z-axis allows the delivery system to maintain the set distance from the surface of the wound to accommodate the curvature of patient’s body. Reconstructing skin wounds can be tailored to an individual patient by combining a wound scanning system with the cartridge-based delivery system (Fig. 1C). To print a skin construct that exactly matches a patient’s wound, the current prototype incorporates a real-time 3D laser scanner, ZScanner™ Z700 scanner (3DSystems, Rock Hill, SC), utilizing markers that are placed around the wound area used as reference points (Fig. 1D). The hand-held scanner is easy to use and allows for easy maneuverability. The scanner offers the ability to capture the entire wound in one continuous scan. Once the scan is complete, the wound data is compiled to form a model of the wound. The scanned wound area is then processed using Geomagic Studio software (Morrisville, NC) orient the model to standard coordinates so the import into Artcam is ideal. Scanned data is in the form of an STL file that has been outputted from Geomagic and inputted to Artcam® 3D software to obtain the full volume and the nozzle path needed to print the fill volume. The wound depth is then split into layers on the Z axis to determine which layers correspond to the dermis and epidermis (Fig. 1E). Each layer is overlaid with a series of XY lines that cover the entire wound area. These lines are used in conjunction with the cartridge-based delivery system to determine a path for the printer. The skin cell delivery system is controlled by a custom software employing a three-tiered architecture design based on the Microsoft.NET Framework 2.0 (Microsoft Corp^®^. Redmond, WA) and written in C++. The three-tiered architecture handles three areas of communication: between the user and the software, among the software components, and between the software and the file system. This design structure allows every component to be quickly and easily altered as necessary without affecting the other components.

The cartridge-based delivery system is similar to that used in traditional inkjet printing. The pressure-based delivery system uses three components: a delivery mechanism, the material reservoirs and a pressure source. The delivery system is based on cartridges that can contain any material that can be delivered through the print-head nozzles, with each nozzle connecting to a separate cartridge. For the skin delivery system the contents of each cartridge are embedded in a matrix composed of fibrinogen and collagen. Separate atomizing nozzles simultaneously deposit thrombin on the fibrinogen matrix to produce fibrin. Each cell type is loaded into an individual cartridge in the same way different color inks would be contained in different cartridges. For our clinical printer, design updates were performed to incorporate a more controllable piston-driven pressure mechanism. For the air-pressure system, air from the pressure source fills the reservoir and drives the material in the reservoir into the delivery mechanism as it is needed. The system is pressurized with 6.89 kPa of compressed air using a two-stage control valve. The piston-driven pressure delivery was achieved with individual syringe pumps, and was able to achieve identical pressures but with more control over ramping and depressurization. Both systems resulted in identical functional outcomes. Together, these components provide a powerful new tool for treatment of large wounds.

### Bioprinting Procedure

The sterilization, preparation and bioprinting procedures are easy and quick to perform. The system is designed to be covered with a sterile drape. The print-head is detachable and able to be autoclaved. Sterilization of tubing was performed by connecting cartridges of 70% ethanol followed by nano-filtered autoclaved water to clear the residual ethanol. A sterilization command directs the printer to automatically flush the delivery system with ethanol for 3 minutes followed by a sterile water flush for 1 minute. The user can choose to print using one of three different methods. First, the laser scanner can be used as previously described to create a wound map for printing. Second, the user can define a series of bitmap images where each non-black pixel corresponds to a cell drop, and the color of the pixel corresponds to the cell type to print. This method allows the user to create complex structures using any cell type in any configuration. Finally, a list of available cell types loaded in the printer is provided to the user by the print-head configuration. In each method, the user defines the spacing between drops, and this is only limited by the resolution of the motors in the plotting system. A laser sight guided the user to the upper-left corner of the wound and the distance from the laser to the first inkjet valve was used as a calibration that ensured accurate and repeatable delivery of cells. The fibrin/collagen hydrogel carrier used in this study provides suitable support to maintain cellular viability, rapid crosslinking, and accurate depositing to facilitate the formation of multicellular, multi-layered skin constructs. In these studies the integrated scanning system enabled us to rapidly measure and convert wound size, depth and topology into a two layered format comprising of a lower fibroblast layer with an upper keratinocyte layer.

### Bioprinter Validation in a Murine Full-Thickness Wound Model

**Figure 2:**
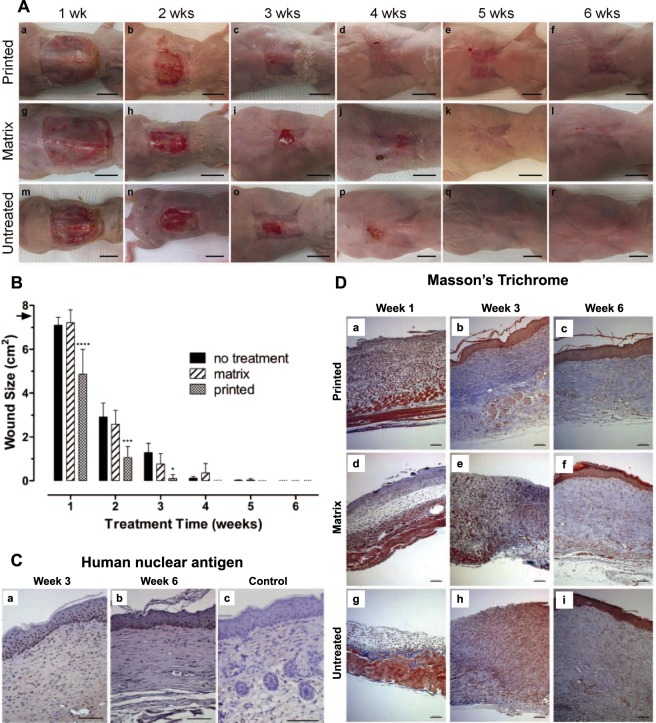
Gross examination of printed skin in a murine full thickness excisional wound model. (**A**) Cell printed mice show epithelium forming over the wound as early as week 1, with developing skin observed at week 2 that covers the entire wound but has not fully formed. By week 3, cell printed mice show complete coverage of the wound. Between week 4 and week 6, minimal contraction is observed and we observe a maturing epithelium. In contrast, matrix-treated and untreated wounds show minimal epithelialization until week 4, resulting in a significant proportion of open wound area. Contraction is also significant between weeks 4 and 6 following closure of the wounds. Scale bar: 1 cm. (**B**) Analysis of murine wound sizes over 6 weeks. Printed skin constructs show a significantly reduced time to wound closure when compared to untreated and matrix-treated controls. Printed skin closed the wound in 3 weeks compared to 5 weeks for controls. Wound sizes were analyzed with one-way ANOVA. ****p < 0.0001, n = 12; ***p < 0.01, n = 8; *p < 0.05, n = 8. Data presented as mean ± standard deviation (SD). (**C**) Anti-human nuclear antigen showed printed skin at the center of the wounds contained human within the epidermis and dermis at week 3, and week 6 after bioprinting. Matrix printed control wounds showed no human cells present at 6 weeks. Scale bar: 100 µm. (**D**) Masson’s Trichrome examination of skin. (a–c) Printed skin at the center of the wound shows increased cellularity compared to the matrix-treated and untreated controls, but lacked the presence of a defined epidermis or dermis maturation. At week 3 and week 6 post-printing we observed the presence of a defined epidermis and organized dermis consisting of aligned blue stained collagen fibers. (d–f) Matrix-printed wounds lacked cellularity and presence of a defined epidermis or dermis maturation one week after printing. At week 3 after treatment, matrix-printed still lacked a defined epidermis and showed no dermal organization but started to show epidermis formation and a more defined dermis with increased prominence of blue stained collagen fibers by week 6 (g–i) Untreated wounds lacked both the cellularity and material thickness observed in the other two groups at week one, but appeared similar to matrix only-treated wounds at week 3 and 6. Scale bar: 100 μm.

We first validated the *in situ* skin bioprinting system using a murine full thickness excisional wound model. In the 36 female outbred athymic nude (Nu/nu) mice used in this study, the mortality rate was 0% and no wound infection or major skin irritation was noted on any mouse at any time point. Gross examination revealed that every printed skin construct appeared to form an epithelial coverage over the wound at 1 week (Fig. 2A). This epithelium became more organized with time until the wound was completely covered. Organization into fully-formed skin required approximately 10–14 days post-printing.

Wounds treated using *in-situ* skin bioprinting demonstrated faster wound closure compared to untreated and matrix-treated group (Fig. 2B). In the printed group, the wound area at 1 week post-surgery was 66% of the original wound area. In contrast, the wounds in the control groups remained at 95% of their original wound size at the same time point (n = 12, p ≤ 0.001). At 2 weeks, the wounds treated with printed cells were only 15% of the original size, while the wounds in the control groups were 40% of the original size (n = 8, p ≤ 0.05). Overall, printed skin cells were able to close the entire wound by 3 weeks post-surgery compared to 5 weeks for both negative controls.

Immunohistochemistry for human cells showed that human fibroblasts and keratinocytes were present within the dermis and epidermis of the wound 3 weeks and 6 weeks after printing, together with endogenous cells (Fig. 2C). Control groups did not show any staining for human cells. Histological examination of retrieved skin constructs revealed high cellularity of printed wounds one week after printing (Fig. 2D). This was expected, as our system delivers approximately 500 cells per drop using the current prototype. This histological finding supports the observation of epithelialization of the bioprinted wounds, and indicates that cells were present in the wound, perhaps forming an immature epidermal barrier. At week 3 and week 6 post-printing we observed the presence of a defined epidermis and organized dermis consisting of aligned blue stained collagen fibers. This finding is consistent with the accelerated wound closure and re-epithelialization. In matrix-printed wounds, the deposited hydrogel was visible within the wound area one week after printing, but as expected, lacked cellularity. At week 3 after treatment, matrix only-treated wounds showed increased cellularity, suggesting migration of endogenous cells, however these wounds lacked a defined epidermis and showed minimal dermal organization. At week 6 after treatment matrix-only treated wounds started to show epidermis formation and a more defined dermis with increased prominence of blue stained collagen fibers. Untreated wounds lacked both the cellularity and material thickness observed in the other two groups at week one, but appeared similar to matrix only-treated wounds at week 3 and 6 In all printed constructs the dermis contained a highly organized deep reticular layer and a less-organized papillary layer. The major three layers of the skin were present. All groups showed complete wound closure and healing by the end of week 6.

### Bioprinter Validation in a Porcine Full-Thickness Wound Model

**Figure 3:**
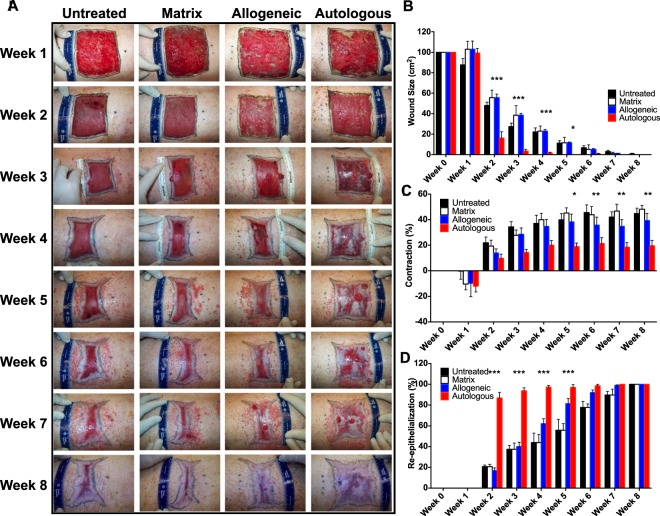
Gross examination of printed skin in porcine model. (**A**) Images of wound healing of *in situ* bioprinted autologous and allogeneic fibroblasts and keratinocytes compared to bioprinted fibrinogen/collagen (matrix only) and untreated control groups over 8 weeks. Formation of epidermis islands at the core of the autologous treatments started as early as week 2 and complete wound closure and re-epithelialization with minimal contracture was achieved by week 4 of the study. All other treatments appeared to contract significantly, with little epithelium forming primarily from the edges of the wound. (**B**) Significantly smaller open wound size was measured for autologous cell-treated wounds compared to other treatments. (**C**) Wound contraction was also reduced in autologous cell-treated wounds compared to other treatments. (**D**) Autologous cell-treated wounds showed significantly accelerated re-epithelialization compared all other groups. Wound sizes were analyzed with one-way ANOVA. ***p < 0.001, n = 6; **p < 0.01, n = 6; *p < 0.05, n = 6. Data presented as mean ± standard deviation (SD).

An overview of the porcine model study design is shown in Supplementary Fig. S1. All wounds formed granulation tissue by week 1 following excision, with greater levels of granulation appearing in the matrix, allogeneic and autologous cell-treated wounds (Fig. 3A). By week 2 we observed granulation tissue still apparent in the allogeneic and autologous cell-treated wounds, but to a lesser extent in the matrix-treated wounds. At this time, we also observed the beginning of contraction in the untreated wounds, with tattoo borders beginning to reduce in size and curve inwards, presenting a typical contractile wound shape. Autologous cell-treated wounds appeared to form a thin epithelium over the majority of the wound area by 2 weeks, which then thickened and became white opaque from 4 weeks onwards. Interestingly, these opaque, white areas of epithelium originated from small patches within the wound area, and grew throughout the time-course of the experiment, suggesting that these epithelial patches did not originate from the tissue edge but rather from autologous cells applied with the bioprinter. During weeks 2 to 4, autologous cell-treated wounds contracted very little, while all other treatments appeared to contract significantly, becoming reduced in total size, but also losing the square shape and becoming rectangular with pointed corners, suggesting contraction toward the center of the wounds. These trends continued between weeks 4–8, with autologous cell-treated wounds showing increased opaque, white-appearing epithelium, while maintaining tissue shape and size. In contrast other groups continued to contract, with little epithelium forming primarily from the edges of the wound. Between weeks 6 and 8 it appeared that all wounds had reached their final contracted size.

Wound size was measured for each wound once per week and recorded as area in centimeters squared (cm^2^) (Fig. 3B). One week following surgery, most wounds maintained their original size, however, contraction did occur in the untreated wounds, resulting in a decrease in wound size to a mean of 87.75 ± 15.25 cm^2^ (p < 0.05). From this time-point onwards, untreated wounds were statistically indistinguishable in size from matrix, and allogeneic cell-treated wounds, although the trend was for a smaller wound size at weeks 1 to 3. From week 2, 3, 4 and 5 autologous cell-treated wounds closed rapidly, and open wound area as significantly smaller than all other groups. Specifically measuring: 16.14 ± 15.29 cm^2^ at week 2 (p < 0.001 vs all groups), 3.40 ± 4.64 cm^2^ at week 3 (p < 0.001 vs all groups), 1.46 ± 2.09 cm^2^ at week 4 (p < 0.001 vs all groups), and 0.00 ± 0.00 cm^2^ at week 5 (p < 0.05 vs all groups). From this point onwards other treatments also trended in this direction, with no statistical differences between groups. These data suggest that the *in situ* bioprinting of autologous cells resulted in approximately 3-week acceleration in wound closure compared to other treatments.

Wound contraction was measured for each wound at the wound tattoo border once per week and expressed as a percentage of the original wound tattoo area (Fig. 3C). One week after treatment, all wounds other than untreated expanded in size, resulting in a negative contraction value. Untreated wounds only showed minimal expansion. This effect is observed in many of our excisional wound models, and represents a combination of sagging or stretching of the wound area following full thickness excision, with some contribution from swelling and granulation. By week 2, this trend was reversed with all groups showing positive contraction (shrinking), without statistically significant differences between groups. At week 3 autologous cell-treated wounds were significantly less contracted than untreated wounds (14.33 ± 5.99% vs 34.3233 ± 10.24%, p < 0.05), and at week 4 autologous cell-treated wounds were significantly less contracted than matrix-treated wounds (20.00 ± 8.96% vs 39.91 ± 12.42%, p < 0.05). However compared to allogeneic-treated wounds there were no statistical differences at these time-points, although the obvious trend for the duration of the study was reduced contraction in autologous cell-treated wounds. From weeks 5 to 8, autologous cell-treated wounds showed significantly less contraction than both untreated and matrix-treated wounds (week 5, p < 0.05; week 6–8, p < 0.01) and also less contraction than allogeneic cell-treated wounds (p < 0.05). These data suggest that bioprinting autologous cells to full thickness wounds resulted in approximately 50% reduction in wound contraction compared to other treatments over the time-course of the study.

Wound re-epithelialization was expressed as a percentage of total area within the tattoo border covered by epithelium (Fig. 3D). No epithelialization was recorded at week 0 or week 1 for any group. As early as week 2, autologous cell-treated wounds showed significantly greater percentage of re-epithelialization compared to all groups between weeks 2 and 5. Specifically measuring: 86.83 ± 13.30% at week 2 (p < 0.001 vs all groups), 93.73 ± 7.12% at week 3 (p < 0.001 vs all groups), 97.24 ± 4.23% at week 4 (p < 0.001 vs all groups), and 97.00 ± 6.71% at week 5 (p < 0.001 vs all groups). At week 6, autologous cell-treated wounds showed significantly greater percentage of re-epithelialization than both untreated and matrix-treated wounds (98.57 ± 3.50%, p < 0.05), but this was not significantly different to allogeneic cell-treated wounds (92.11 ± 6.17%). Similar to the wound closure data, from this point onwards, autologous cell-treated wounds showed complete wound closure and re-epithelialization and other treatments trended in this direction, with no statistical differences between groups. These data show that bioprinting autologous cells to full thickness wounds resulted in rapid epithelialization of the wounds, representing a 4–5 week acceleration in wound re-epithelialization compared to other treatments.

### Porcine Wound Histological Analyses

**Figure 4:**
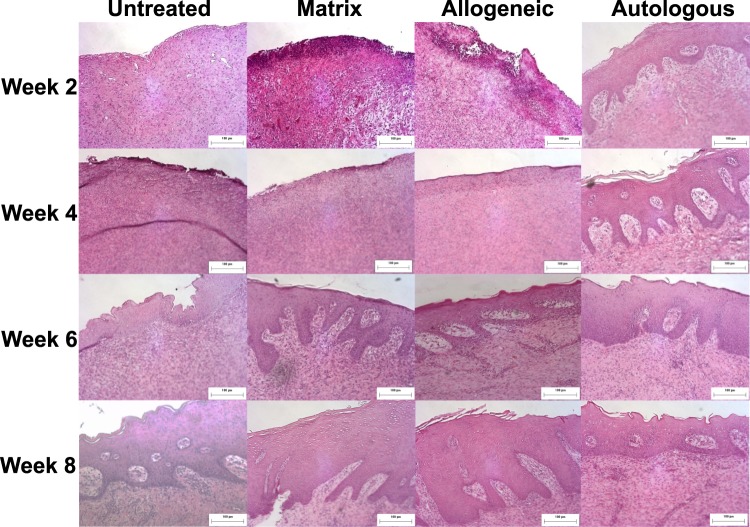
Microscopic examination of H&E stained sections showed increasing degrees of tissue regeneration, epithelialization and maturation as wound healing progressed. Wounds receiving bioprinted autologous cells showed early epithelialization with almost complete coverage of the wound at 2 weeks. By week 4, autologous bioprinted wounds showed the formation of rete peg epithelial projections into the dermis, as well as the presence of keratinized stratified squamous epithelium. Wounds treated with bioprinted autologous cells showed early formation of a loosely organized papillary layer above a denser, thicker layer of reticular dermis. The structure of the tissue appeared mature and complete at 8 weeks with nicely woven dermal collagen and regularly distributed vasculature. Magnification 20x, Scale bars 100 µm.

Microscopic examination identified increasing degrees of tissue regeneration, epithelialization and maturation as wound healing progressed through the weeks (Fig. 4). All wounds progressed through an initial formation of granulation tissue on the surface followed by a thin discontinuous layer of squamous epithelium that eventually became continuous and covered the entire wound. Wounds receiving bioprinted autologous cells showed early epithelialization with almost complete coverage of the wound at 2 weeks. By week 4, autologous bioprinted wounds showed the formation of rete peg epithelial projections into the dermis, as well as the presence of keratinized stratified squamous epithelium. In contrast, allogeneic cell and matrix-treated wounds did not show the presence of epithelialization or keratinized stratified squamous epithelium until week 6. Untreated wound healing appeared even more delayed, with only an immature epithelium present at week 6, which still lacked the mature-appearing rete peg epithelial projections and keratinized stratified squamous epithelium at week 8.

Wounds treated with bioprinted autologous cells showed early formation of a loosely organized papillary layer above a denser, thicker layer of reticular dermis. The papillary dermis appeared to consist of coarse bundles of larger loose fibers arranged parallel to the epidermis, while the reticular dermis contained larger blood vessels and closely interlaced smaller fibers. The structure of the tissue appeared mature and complete at 8 weeks with nicely woven dermal collagen and regularly distributed vasculature. Allogeneic cell and matrix-treated wounds showed the continued presence of granulation tissue 2 weeks after treatment, progressing to a dermis consisting of a densely packed fibroblastic proliferation lacking distinction between the papillary and reticular dermal layers at week 4. By weeks 6 and 8 the dermis in these groups appeared similar to the autologous cell-treated group in appearance, matching the wound healing progression time frame observed for the epidermis. Untreated wounds generally lacked the granulation tissue formation observed histologically for other groups, showing only a densely packed dermis and lacking blood vessel formation. This continued until week 6, where the dermis matured at a similar, but slower progression than seen with other groups.

It is well known that the more prolonged the inflammatory phase of wound healing is, there is more likelihood of generating a non-functional scar. Therefore, we focused on assessing the degree and types of inflammation histologically. A pathologist blinded to the treatment groups scored acute and chronic inflammation in H&E stained sections using the grading criteria shown in Supplementary Fig. S2. Acute inflammation was most abundant in week 2 samples of all groups and decreased over the wound healing progression (Table 1). Acute inflammation was mostly superficial and appeared to be inversely related to the degree of epithelialization. It was very prominent in all groups at 2 weeks (score of 3) except for the autologous cell-treated wounds, which only showed mild acute inflammation (score of 1). Acute inflammation was mostly absent or very mild by the 8th week in all groups. Chronic inflammation was present in all wounds and it was mainly in the deeper aspects of the wound. It persisted throughout the study duration and did not appear to affect maturation and re-epithelialization of the wound. Granulomatous inflammation and foreign body giant cells were noted in the deeper aspects in some of the grafts with no noticeable association with any specific treatment type.

**Table 1:** Grading of acute and chronic inflammatory response of treatments.

Time Point	Inflammation	Untreated	Matrix Only	Allogeneic	Autologous
2 weeks	Acute	3	3	3	1
	Chronic	0	2	2	2
4 weeks	Acute	2	2	3	1
	Chronic	1	1	2	1
6 weeks	Acute	1	2	1	0
	Chronic	2	1	2	1
8 weeks	Acute	1	0	1	0
	Chronic	3	2	2	2

Autologous cell-treated wounds showed minimal signs of acute inflammation at 2 weeks; whereas, the untreated, matrix-treated and allogeneic cell-treated wounds showed dense neutrophilic inflammation and granulation tissue on the surface of the wound at this stage. Chronic inflammation of autologous treatments was less or comparable to other treatments. Chronic inflammation may be associated more with the surgical intervention procedures and not to the treatments.

**Figure 5:**
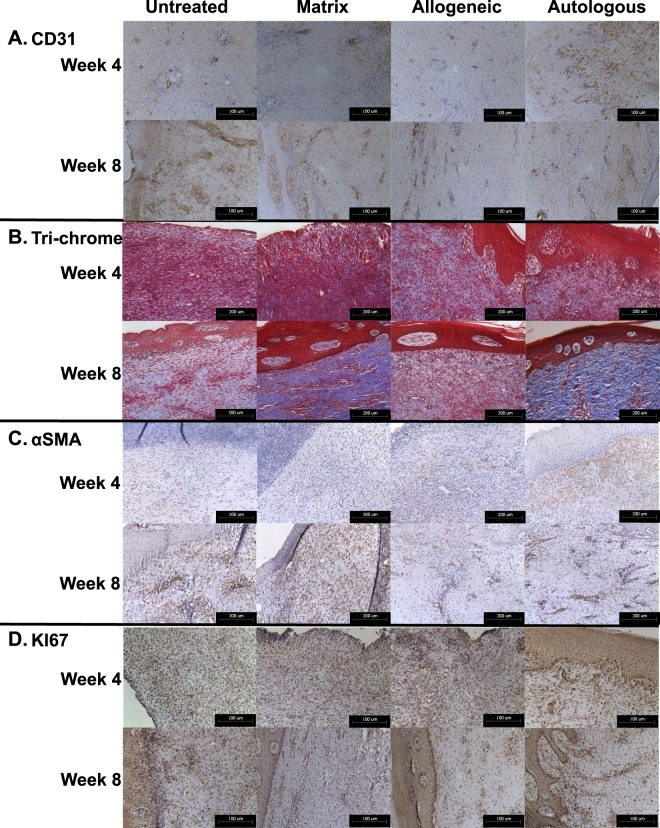
Histological evaluation of vascularization, collagen deposition, myofibroblast activation, and cell proliferation. (**A**) We observed a higher density of CD31-positive blood vessels throughout the dermis of autologous cell-treated wounds at week 4, transitioning to larger mature vessels throughout the dermis at week 8. (**B**) Blue stained collagen fibers were most prominent in matrix-treated and autologous cell-treated wounds. Collagen fibers present in the autologous cell-treated wounds appeared much larger and organized than observed in other groups. (**C**) αSMA-positive cells were more prominent in the autologous cell-treated wounds at week 4. At week 8, the untreated and matrix-treated wounds showed significantly greater numbers of αSMA-positive cells, distributed throughout the dermis, and surrounding the sparse blood vessels, suggesting the presence of large numbers of contractile myofibroblasts at this time-point. (**D**) At week 4, untreated, matrix and allogeneic cell-treated wounds showed the greatest number of proliferating cells, primarily located throughout the dermis. Autologous cell-treated wounds showed fewer overall proliferating cells, however these cells were mostly present immediately underlying the developing epidermis, suggesting that these cells were keratinocytes contributing to epidermis formation and maturation. CD31/Ki67: Magnification 20x, Scale bars 100 µm, Trichrome/αSMA: Magnification 40x, Scale bars 200 µm.

To evaluate the formation and maturation of wound vasculature, CD31 immuno-staining was performed at weeks 4 and 8 of wound regeneration (Fig. 5A). Concurring with our observations of early granulation tissue formation in the autologous cell-treated wounds, we identified a higher density of CD31-positive blood vessels throughout the dermis of these wounds, with only minimal, small blood vessels sparsely distributed throughout the dermis of the other groups. By week 8, the overall number of small blood vessels appeared to decrease in autologous cell-treated wounds, and instead we observed larger mature vessels throughout the dermis. In contrast, untreated wounds showed large numbers of small, immature blood vessels, suggesting that these tissues were undergoing delayed wound healing, appearing similar to autologous cell-treated wounds at week 4. Matrix and allogeneic cell-treated wounds lacked the high density of small blood vessels observed in the week 4 autologous, and week 8 untreated wounds, and instead showed a reduced number of mature blood vessels.

Masson’s trichrome staining of tissue sections at week 4 highlighted a dermis composition with densely packed nuclei, potentially due to inflammatory cells, and generally lacking organized collagen fibers (Fig. 5B). This was most prominent in the untreated and matrix treated groups. Allogeneic and autologous cell-treated groups showed more diffuse nuclei distributed in a background of organized collagen fibers. At week 8, the concentration of cell nuclei was significantly reduced in all groups, however patches of concentrated cells were still present in the dermis of the untreated, matrix-treated and allogeneic cell-treated groups. These were still present but fewer in number in autologous cell-treated groups. Blue stained collagen fibers were most prominent in matrix-treated and autologous cell-treated wounds. Interestingly this was not observed to the same extent in allogeneic cell-treated wounds. Collagen fibers present in the autologous cell-treated wounds appeared much larger and organized than observed in other groups, supporting histological observations that the dermis of these wounds appeared more mature than for other treatments.

Fibroblastic differentiation into myofibroblasts plays a major role in the formation of wound granulation tissue early in wound healing, and persists in contractile wounds, such as hypertrophic scars. To evaluate the presence of myofibroblasts in the healing wounds, we performed immuno-histochemistry for α-smooth muscle actin (αSMA) at weeks 4 and 8 (Fig. 5C). αSMA-positive cells were largely absent from untreated and matrix-treated wounds at week 4, while the allogeneic and autologous cell-treated wounds showed moderate staining in the papillary region of the dermis. This staining was more prominent in the autologous cell-treated wounds. At week 8, the untreated and matrix-treated wounds showed significantly greater numbers of αSMA-positive cells, distributed throughout the dermis, and surrounding the sparse blood vessels, suggesting the presence of large numbers of contractile myofibroblasts at this time-point. In the allogeneic and autologous cell-treated wounds, αSMA staining was primarily located around the larger mature blood vessels, supporting observations of increased blood vessel formation and maturation in these tissues.

Ki67 immuno-staining was performed at weeks 4 and 8 to evaluate the presence of proliferating cells within the wounds (Fig. 5D). At week 4, untreated, matrix and allogeneic cell-treated wounds showed the greatest number of proliferating cells, primarily located throughout the dermis, potentially activated myofibroblasts, and at the wound surface, which had not yet formed an immature epidermis. Autologous cell-treated wounds showed fewer overall proliferating cells at week 4, however these cells were mostly present immediately underlying the developing epidermis, suggesting that rather than proliferating myofibroblasts, these cells were keratinocytes contributing to epidermis formation and maturation. At week 8, the untreated, matrix and allogeneic cell-treated wounds showed a similar staining pattern to that observed in the week 4 autologous cell-treated wounds, while the week 8 autologous cell-treated wounds showed an even greater number of proliferating cells in the underlying epidermis area.

### Skin bioprinting comparison with cell spraying

We evaluated the efficacy of our bioprinting technique and compared to a cell spraying technique, which is becoming commonly applied in the clinic for treatment of wounds using autologous skin cells. Specifically, we compared our *in situ* skin bioprinting system using the printing parameters described above, to the FibriJet biomaterial delivery device using the same materials, concentrations, volumes and application timing for both techniques. Similar to our previous studies, both groups formed granulation tissue by 2 weeks followed by rapid wound closure through a combination of contraction and re-epithelialization, resulting in total wound closure (Supplementary Fig. S3A). Although the bioprinted wounds appeared to show more rapid wound closure, reduced contraction and increased re-epithelialization, there were no statistical differences for these parameters at any time-points (data not shown). This suggests that for these parameters, our bioprinting system is equally as effective as the currently available clinical cell spraying technologies.

Interestingly, histological analysis of the wounds at each time-point showed that the bioprinted wounds resulted in earlier epithelialization with almost complete wound coverage at 2 weeks, while the cell sprayed wounds did not show the formation of epidermis until week 4 (Supplementary Fig. S3B). In fact, the epidermis of the cell spray groups did not thicken and showed signs of maturation such as presence of keratinized stratified squamous epithelium until 6 weeks. Wounds treated with the bioprinted cells showed early formation of a papillary layer consisting of coarse bundles of larger loose fibers, distinct from a dense, thicker layer of reticular dermis containing larger blood vessels and closely interlaced smaller fibers. In contrast, wounds treated with the sprayed cells showed a dermis consisting of a densely packed organization lacking a distinction between the papillary and reticular dermal layers at week 2, which eventually matured to form a more distinct and mature dermis similar to bioprinted wounds by week 4. Trichrome staining supported these observations, confirming that the bioprinted wounds showed accelerated formation of epidermis and more mature dermis tissue at weeks (Supplementary Fig. S3C). Collagen fibers stained in blue were more prominent in the bioprinted wounds at week 4, compared to wounds treated with the sprayed cells. By week 6, both groups had similar appearance with a defined epidermis and organized dermis, consisting of aligned collagen fibers.

## Discussion

This study describes the proof-of-concept validation of a mobile *in situ* skin bioprinting system with integrated imaging technology to provide rapid on-site management of full thickness wounds. We found that treatment with autologous fibroblasts and keratinocytes, delivered directly to specific locations of the wound based on wound size and topology, resulted in the acceleration of wound healing and the formation of normal skin *in situ*.

Other groups have similarly demonstrated that bioprinting can be a useful tool for the fabrication of skin tissue substitutes for wound healing. Lee *et al*. demonstrated the use of a solid freeform robotic system with noncontact dispensers capable of distributing keratinocytes and fibroblasts at a high cell density at high resolutions^30^. This bioprinted skin tissue was morphologically and biologically representative of native human skin tissue. Another group used the laser-assisted bioprinting to fabricate a 3D arrangement of fibroblasts and keratinocytes as a multicellular graft analogous to native skin archetype^31^. These skin constructs appeared to demonstrate tissue formation following implantation into a nude mouse model^32^. Yet, another group has developed a modified extrusion bioprinter to deposit a combination of human plasma, human fibroblasts, calcium chloride and human keratinocytes. The bioprinted construct was polymerized and grafted onto immunodeficient mice for up to eight weeks after which it was indistinguishable from bilayered dermo-epidermal equivalents^33^.

Our bioprinter system has combined an inkjet based bioprinter technology capable of rapidly depositing materials and cells with high precision, with wound scanning imaging technology to specifically measure wound topology, and allow the precise and accurate delivery of appropriate cell types to specific areas of the wound represents a significant advance in personalized wound treatment approaches. In this study, we first performed proof-of-concept studies using the murine model to demonstrate our system was able to provide immediate and proper coverage of wounds. We then demonstrated the capabilities of our system to deliver allogeneic or autologous dermal fibroblasts and epidermal keratinocytes within a biological hydrogel to large full thickness wound in the porcine model. Wound treatment using our bioprinting system resulted in the quick and proper coverage of the wounds, which is crucial for maintaining homeostasis, wound closure, epithelialization and scar prevention.

We demonstrated a proof-of-concept of our system by printing a bilayered skin construct consisting of human fibroblasts and keratinocytes directly onto a full-thickness skin defect on a nude mouse. These studies demonstrated the capabilities of our bioprinting system to deliver appropriate cell types and concentrations in a layered manner. Evaluation of the wound area over a 6-week period showed rapid closure of the wound in the printed mice compared to the untreated and matrix controls. Although murine wound healing studies represented a cost-effective and rapid proof-of-concept for our skin bioprinting system, significant differences in murine and human skin healing rates and mechanisms necessitated a more suitable porcine wound model for further evaluation.

In our porcine studies, wounds treated with autologous fibroblasts and keratinocytes performed better in every outcome evaluated. Autologous cell-treated wounds showed accelerated wound closure, reduced wound contraction and increased re-epithelialization. These wounds also showed an acceleration of health skin formation and maturation, with histological evaluation showing increased formation of granulation tissue followed by the development and maturation of a complete epidermis. Pathologic examination correlated very well with gross appearance of the wounds, with rapid formation of new epithelium. It also reinforced the common understanding that granulation tissue formation is an essential first step in wound healing [30]. These tissues showed a dermal structure and composition similar to healthy skin, with extensive collagen deposition arranged in large, organized fibers, extensive mature vascular formation and high numbers of proliferating keratinocytes. These factors all support the conclusion that this treatment results in the acceleration of wound healing through the formation of healthy and functional skin *in situ*. These effects were not observed for the other treatment groups, with matrix only-treated wounds and allogeneic cell-treated wounds appearing similar to untreated wounds. While some minor differences were observed for some outcome measures, such as formation of granulation tissue and dermal collagen deposition, these differences did not seem to significantly impact wound healing or skin development. Based on these findings, it appears that the biomaterials used to deliver cells to the wound did not contribute, negatively or positively, to wound development.

Similarly, the inclusion of allogeneic cells did not appear to provide any benefit to wound closure or healing. While this is not unexpected, as eventual rejection of cells is likely to occur in immune-competent animals, it does highlight a limitation of this method for treating patients with skin burns or wounds covering the majority of the body, and where biopsy sites may not be available. Additionally, while a short period of culture and expansion of autologous cells has been shown to be advantageous for wound healing applications^21^, many wounds require rapid treatment which may prevent the ability to culture the isolated cells, reducing cell number and efficacy of the treatment. This is of key importance, as our preliminary studies suggests that wound healing time is directly proportional to the number of autologous cell delivered to the wound (unpublished data). Strategies to overcome this limitation may include the development of HLA-matched skin cell banks, the use of stem cells with potential immune-privileged status, or novel methods to increase cell isolation efficiency and functional activation prior to printing.

This study was mainly focused on the rate of wound healing and the ability to cover large wound areas. The efficacy of our bioprinting technique was compared to a cell spraying technique commonly used in the clinic, where cells are retrieved from a blend of autologous tissues and sprayed as a mixture. Other than the different methods by which the materials and cells are delivered to the wounds, the main difference between these two techniques is that the bioprinting approach delivers epidermal and dermal cells that are dispensed separately in a layer-by-layer fashion, rather than an unorganized mixture of both cell types. We acknowledge that a common clinical application of cell spraying often involves the delivery of autologous cells without culture expansion in a point-of-care clinical setting, however in order to facilitate a direct comparison of the bioprinting and cell spraying, we used culture expanded cells as used in previous bioprinting studies, as well as maintained identical biomaterial and cell concentration, volumes and treatment protocols. In this comparative study, we did not observe significant differences between the two techniques in regard to measurements of wound closure, contraction or re-epithelialization. The bioprinted wounds appeared to show accelerated closure, increased epithelialization and reduced contraction. However, where we observed noticeable differences was in the early formation of a defined epidermis and mature dermis layers. This observation may be explained by one or more influencing factors. First, it is likely that the cell-spraying device may have exposed cells to an instant, high-pressure and flow rate at the spray nozzle, negatively affecting their viability and function. A more likely explanation for this observation is that the *in situ* bioprinting system enables the dermal fibroblasts and epidermal keratinocytes to be dispensed in a spatial orientation that matches the architecture of the wound. Such a system may provide multiple layers with more cell types specific to each skin level (epidermis, dermis, fatty subcutis, hypodermis) to promote integration and formation of vascular and neural networks for the regeneration of functional skin. Further studies are necessary to elucidate the reasons for these observed differences, however these data suggest that our skin bioprinting technology has the potential to improve wound treatments compared to currently used cell delivery technologies.

The duration of the study does not allow for assessment of long-term skin function and cosmetic results. However, given the significantly reduced wound contraction and the histomorphologic evidence of well-organized tissue with proper vascularity and limited inflammation, we would expect reasonable cosmetic results on the long term. Additionally, the short time-period for cell isolation and culture limited our ability to perform suitable cell tracking experiments. Lentivirus-labeling of isolated primary cells with a long-lasting fluorophore was not feasible due to the need for selection of labeled cells, which would further decrease cell yield. The use of membrane-bound fluorescent dyes were found to fade rapidly due to *in situ* cell proliferation, limiting their usefulness for the 8 week time course of this experiment. Unfortunately, these limitations do not allow us to conclusively determine whether bioprinted cells were cleared from the wounds, or what proportion of the regenerated skin consist of bioprinted cells. However, it is clear from these studies that *in situ* bioprinting of autologous cells resulted in the rapid formation of new skin, preventing contraction and accelerating the formation of healthy tissue structure and function.

Our future studies will utilize this printing system to investigate the delivery of other biomaterials and cell types to further accelerate wound healing, reduce the requirement for biopsies and cell culture, and potentially provide an off-the-shelf cartridge that can be used to rapidly treat patients either whether they are in civilian hospitals or military field hospitals. The inclusion of additional skin cell types is currently being evaluated, with the inclusion of melanocytes, adipose cells, and hair follicle cells promising to provide the rapid formation of skin tissue with superior functional and cosmetic outcomes. This technology has a wide application, and its application will be expanded to not only treat full-thickness wounds, but also for other types of wounds such as burns and ulcers, and even for deep tissue injuries.

## Materials and Methods

### Experimental Design

This study was designed as a controlled laboratory experiment for the proof-of-concept validation of a mobile *in situ* skin bioprinting system with integrated imaging technology to provide rapid on-site management of full thickness wounds. Sample sizes for each of the experiments were calculated using power analysis to ensure appropriate study power for our anticipated differences in means and standard deviations. For mouse studies 36 female outbred athymic nude (Nu/nu) mice, divided into three groups of 12 animals in each treatment group (n = 4 per time-point). For porcine studies, six animals were used for the bioprinting study (n = 6 per treatment). Data collection was performed at predetermined time-points as described below. Outliers were defined as data-points that were greater than two standard deviations from the mean, and none were identified in these studies. Study endpoints were defined prior to study commencement and included improved wound closure, re-epithelialization and contraction as primary endpoints, and healthy, mature skin formation as determined by histology as a secondary endpoint.

### Materials

Fibrinogen from bovine plasma Type I-S, 65–85% protein (≥75% of protein is clottable) and thrombin from bovine plasma lyophilized powder, 40–300 NIH units/mg protein were purchased from Sigma Aldrich (St. Louis, MO). Collagen I from rat-tail was purchased from BD Biosciences (San Jose, CA). All other reagents were of chemical grade.

### Murine Wound Creation, Bioprinting, and Bandaging

The efficacy of the skin delivery system prototype to deliver viable skin cells directly onto wounds and replace the missing dermis and epidermis was evaluated by creating a 3 × 2.5 cm (L × W) full-thickness excisional skin defect on the dorsa of 36 female outbred athymic nude (Nu/nu) mice (Charles River Laboratories, Wilmington, MA). This study and all animal procedures were performed according to the protocols approved by the Wake Forest University Health Sciences Animal Care and Use Committee. All experiments were performed in accordance with Animal Care and Use Committee guidelines and regulations. The skin was cleaned with iodine and then sterilized with alcohol swaps. The borders of the wounds were marked on the back of the mouse using sterile marker and ruler. The skin was removed surgically using surgical scalpel blade and defect represents a wound of approximately 50% skin surface area on each mouse. Mice were divided into three groups of 12 animals in each treatment group, with each group euthanized for evaluation at either 1 week, 3 weeks or 6 weeks (n = 4). The first group received no treatment and only standard wound dressing was applied to cover the wounds (untreated), the second group received fibrin and collagen solution alone (matrix), and the third group was treated by printing a layer of human fibroblasts followed by another layer of human keratinocytes (printed). The hydrogel carrier consists of a mixture of 25 mg/ml fibrinogen and 1.1 mg/ml collagen. The pH was adjusted to 7.2 and the solution was syringe filtered using 0.2 µm filter. For the matrix group, the first layer was constructed by printing 0.5 ml of fibrinogen/collagen solution without cells followed immediately by printing 0.5 ml of 20 IU/mL thrombin (extracted from bovine plasma, Sigma Aldrich, St. Louis, MO) to crosslink the fibrinogen and form a fibrin gel. The thrombin was allowed to react before a second layer was printed in the same fashion. Drops were printed at 1 mm interval. For the printed group, 1.875 × 10^6^ fibroblasts were suspended in 0.5 mL of fibrinogen/collagen solution and printed directly onto the wound followed by printing of an equal amount of thrombin. After 15 minutes, 3.75 × 10^6^ keratinocytes suspended in 0.5 mL of fibrinogen/collagen solution was printed on the top of the fibroblast layer. Equal amount of thrombin was printed to form a gel layer. Fibroblasts and keratinocytes were printed at 1:2 cell ratio to mimic the cellular density of in the dermis and the epidermis in the native human skin. After printing of every layer, any remaining cell residue in the printing system was flushed out with water and the system was sterilized using ethanol. Each wound from the three was bandaged by applying triple antibiotic ointment and covered with sterile gauze and surgical tape to prevent bandage damage by the mouse. Dressing was changed every 3 days or as needed under isoflurane anesthesia. Animals were monitored for 6 weeks.

### Murine Wound Analyses

Gross photographic images of wounds were taken every week for 6 weeks. Pictures were taken using digital Kodak camera with 8.2 megapixel lens and a sterile ruler. Wound size measurements were evaluated from these pictures using ImageJ software (National Institute of Health). Wound size was measured by using ImageJ to determine the area of open wound and expressed as percentage of the original wound size. Skin samples retrieved from the mice were fixed in 10% phosphate-buffered formalin overnight at room temperature. Samples were dehydrated in a graded series of ethanol concentrations, embedded in paraffin, and cut into 5 µm sections. Anti-human nuclear antigen slides were washed using buffer consisting of 0.1% saponin in PBS. These slides were incubated in a 1:40 dilution of primary antibody with 0.1% saponin for 60 minutes followed by incubation in a 1:200 dilution of biotin-conjugated horse anti-mouse IgG secondary antibody with 0.1% saponin for 30 minutes. Biotin was labeled with horseradish peroxidase/streptavidin for 30 minutes and visualized with NovaRed. Masson’s trichrome staining was performed to evaluate presence of muscle, collagen fibers, fibrin, and erythrocytes. Briefly, slides were brought to water before submersion in Bouin’s solution, 60 °C for 1 hour, and then Weigert’s hematoxylin for 10 minutes. Sections were then stained for 5 minutes in Biebrich scarlet, rinsed in distilled water and placed in phosphotungstic/phosphomolybdic acid for 10 minutes. Sections were then directly transferred into Aniline blue for 5 minutes, rinsed and then 1% Acetic acid for 1 minute before dehydrating, clearing and cover slipping.

### Porcine Full-Thickness Excisional Wound Creation, and Bioprinting

Specific Pathogen Free (SPF) Yorkshire pigs weighed approximately 40–50 kg at the start of each study. This study and all animal procedures were performed according to the protocols approved by the Wake Forest University Health Sciences Animal Care and Use Committee. All experiments were performed in accordance with Animal Care and Use Committee guidelines and regulations. Six animals were used for the bioprinting study. Animals were sedated with 0.05 mg/kg IM dexmedetomidine and maintained on inhaled isoflurane. They received SC bupivicaine at each wound site. Post-operative analgesics included a transdermal fentanyl patch, famotidine (0.5 mg/kg PO), and carprofen (4 mg/kg PO) for three days. The anesthetized pigs had their backs depilated by shaving. The animals were tattooed with four 10 × 10 cm tattoos on the dorsum to denote the area of the excisional wound. The dorsal skin was cleaned and sterilized with β-iodine and 70% alcohol. For the creation of the defect, 4 areas of skin wound were created by removing 10 × 10 cm of full thickness skin in the central back along the thoracic and lumbar area. Incisions were made along the wound edges with a surgical blade to the panniculus carnosus layer and the overlying skin was excised. After wounds were excised, 10 million fibroblasts were mixed with 6 ml of the fibrinogen/collagen solution as described above and loaded to the skin bioprinter. Thrombin was printed on the top of fibrinogen/collagen solution to form a gel layer. A second layer of keratinocytes was printed on the top of the first layer in the same fashion. Allogeneic fibroblasts and keratinocytes were printed on one wound. Autologous fibroblasts and keratinocytes were printed on a second wound. For matrix only wounds, a total of 12 ml of fibrinogen/collagen solution without cells was printed in two layers in the same fashion described above. One wound remained untreated other than standard bandaging practices used for all wounds.

### Gross Evaluation of Porcine Wound Healing

Gross photographic images of wound were taken every week for 8 weeks. Pictures were taken using digital Kodak camera with 8.2 megapixel lens and a sterile ruler. Wound size, contracture and re-epithelialization measurements were evaluated from these pictures using ImageJ software (National Institute of Health). Wound size and epithelialization was measured by using ImageJ to determine the area of open wound, and epithelium, which was identified by color and texture of the healing wound. Generally open wounds were dark red and shiny, and epithelium ranged from light red and opaque/matte (due to a thin epidermis covering) to white/pink and opaque/matte (due to thicker, mature epidermis). Areas of the wound biopsied for histological analysis were excluded from image analysis, and corrected in total area calculations. Wound size was expressed as percentage of the original wound size and wound re-epithelialization was expressed as percentage of re-epithelialized area compared to the total remaining wound size. Contraction was measured at each of the time-points by measuring the area inside the tattooed square using the software ImageJ and expressed relative to original tattoo size. Wound contraction as expressed as percentage of the original tattoo size.

### Porcine Histological Analyses

Five-millimeter full thickness punch biopsies were taken from the center and the edge of every treatment wound at week 2, 4, 6 and 8 of the study to evaluate wound healing and skin reconstruction. Skin biopsies skin tissues fixed overnight in 10% neutral buffered formalin. Biopsies were then washed in PBS 3 times for 30 minutes per wash, after which the samples were transferred to 30% sucrose for an overnight incubation at 4 °C. Biopsies were then sliced in half, with half processed for paraffin embedding and half flash frozen in OCT blocks in liquid nitrogen. A cryotome or microtome (Leica) was used to generate 4–6 µm sections comprised of the both center and edge portions of the regenerating wounds.

Sections were stained with hematoxylin and eosin for histology, and slides were imaged under light microscopy. Examination criteria included degree of epidermal formation and extent of epithelialization, dermal organization, evaluation of acute and chronic inflammation. Inflammation was evaluated by a pathologist blinded to the treatment groups, using a scale to describe specific levels of acute and chronic inflammation. Masson’s trichrome staining was performed to evaluate presence of muscle/keratin, collagen fibers, and nucleated cells. Briefly, slides were brought to water before submersion in Bouin’s solution, 60 °C for 1 hour, and then Weigert’s hematoxylin for 10 minutes. Sections were then stained for 5 minutes in Biebrich scarlet, rinsed in distilled water and placed in phosphotungstic/phosphomolybdic acid for 10 minutes. Sections were then directly transferred into Aniline blue for 5 minutes, rinsed and then 1% Acetic acid for 1 minute before dehydrating, clearing and cover slipping.

Immunohistochemical (IHC) staining for CD31, α-smooth muscle actin (αSMA) and Ki67 was performed to assess vascularization, myofibroblast differentiation and cellular proliferation, respectively. For IHC, all incubations were carried out at room temperature unless otherwise stated. Slides were warmed at 60 °C for 1 hr to increase bonding to the slides. Antigen retrieval was performed on all slides and achieved with incubation in Proteinase K (Dako, Carpinteria, CA) for 5 min. Sections were permeabilized by incubation in 0.1% Triton-X for 5 min. Non-specific antibody binding was blocked by incubation in Protein Block Solution (Abcam) for 15 min. Sections were incubated for 60 min in a humidified chamber with the primary antibodies CD31/PECAM-1 (1:200, Cat. # MAB33871, R&D Systems), αSMA (1:100, Cat. # ab5694, Abcam), or Ki67 (1:100, Cat. # ab833, Abcam) in antibody diluent (Abcam). Following primary incubation, slides were washed 3 times in PBS for 5 min. Sections were then incubated for 60 min with a biotinylated secondary antibody and peroxidase-labeled streptavidin (LSAB+ System HRP, Dako, Denmark). Stained cells were visualized using the liquid DAB substrate with hematoxylin counterstain.

### Statistical Analyses

Statistical analysis of wound healing data was performed using GraphPad Prism and involved a two-way ANOVA with comparisons between each treatment with each time-point. For multiple comparisons, a Tukey test was used and a significance and confidence interval of 0.05 and 95% respectively. All data is presented as mean ± standard deviation (n = 4 for mouse studies and n = 6 for porcine studies).

## Supplementary information

Supplementary Material

## Data Availability

All materials and reagents are listed in the Supplementary Materials. Further inquiries should be directed to the corresponding author at semurphy@wakehealth.edu.
