# Supplementary material for: In Situ Bioprinting of Autologous Skin Cells Accelerates Wound Healing of Extensive Excisional Full-Thickness Wounds
Source: Sci Rep. 2019 Feb 12;9:1856. doi: 10.1038/s41598-018-38366-w (PMC6372693; doi:10.1038/s41598-018-38366-w)
Supplement: Supplementary file 1 — Supplementary Material [file 41598_2018_38366_MOESM1_ESM.docx]

Title: *In Situ* Bioprinting of Autologous Skin Cells Accelerates Wound Healing of Extensive Excisional Full-Thickness Wounds

**Authors:** Mohammed Albanna^1^*, Kyle W. Binder^1^*, Sean V. Murphy^1^*^†^, Jaehyun Kim^1^, Shadi A. Qasem^2^, Weixin Zhao^1,3^, Josh Tan^1^, Idris B. El-Amin^1^, Dennis D. Dice^1^, Julie Marco^1^, Jason Green^1^, Tao Xu^1^, Aleksander Skardal^1^, James H. Holmes^4^, John D. Jackson^1^, Anthony Atala^1^ and James J. Yoo^1^

**Affiliations:**

^1^Wake Forest Institute for Regenerative Medicine, Wake Forest School of Medicine**,** Medical Center Boulevard, Winston-Salem, NC 27157, USA.

^2^Department of Pathology, Wake Forest School of Medicine**,** Medical Center Boulevard, Winston-Salem, NC 27157, USA.

^3^Jiangsu Key Laboratory of Neuroregeneration, Nantong University, Nantong, China

^4^Department of Surgery**,** Wake Forest School of Medicine**,** Medical Center Boulevard, Winston-Salem, NC 27157, USA.

* Authors contributed equally to this work

^†^ Corresponding author. Email: semurphy@wakehealth.edu

**Supplementary Methods**

***Human Dermal Fibroblast and Keratinocyte Culture***

Human dermal fibroblasts were obtained from human foreskin (ScienCell, Carlsbad, CA) and cultured in High Glucose Dulbecco’s Modified Eagle’s Medium (HG-DMEM, Gibco-BRL, Grand Island, NY) supplemented with 5% fetal bovine serum (FBS) and 1% penicillin/streptomycin (P/S) solution. Human epidermal keratinocytes-neonatal were also purchased from ScienCell and cultured in keratinocyte serum-free media (KSFM) (Gibco-BRL) supplemented with prequalified human recombinant Epidermal Growth Factor 1-53 (EGF 1-53), Bovine Pituitary Extract (BPE) and 1% penicillin/streptomycin (P/S) solution. When sufficient cell numbers were reached in culture, fibroblasts and keratinocytes were trypsinized for 5 min and suspended in the printing solution, which consists of a mixture of 25 mg/mL fibrinogen and 1.1 mg/mL rat tail collagen type I in phosphate buffered saline (PBS).

***Isolation and Expansion of Fibroblasts and Keratinocytes From Split-Thickness Skin Biopsy***

Using a dermatome, one or two split-thickness skin samples measuring up to a total area of 10cm x 10cm were collected and standard bandaging was performed. Skin biopsies were washed twice in 10% antibiotic-antimycotic (ABAM) (Life Technologies, Grand Island, NY) in PBS for 5 minutes each and then in 1% ABAM in PBS for 5 minutes each. Skin biopsies were incubated in a sterile 2.4 units/ml of dispase II produced in Bacillus polymyxa (Life technologies, Grand Island, NY) solution in PBS at 4 °C for 16 hours. Skin biopsies were washed with PBS to remove the trace of dispase. Epidermis was then peeled from dermis using sterile forceps. Fibroblasts were obtained from the dermis. Dermis was cut small strips using sterile surgical blade. Explants were laid down on the culture plates. Explants were covered with DMEM-HG supplemented with 10% FBS and 1% ABAM and incubated at 37 °C in 5% CO_2_ for 4 days to allow fibroblasts to migrate and adhere to the plates. After 4 days, explants were removed from the culture using sterile forceps and fresh media was added. Media was then changed every two days until cells reached 80% confluence. Keratinocytes were obtained by mincing the epidermis using blades and then placed in 0.05% Trypsin-EDTA solution 1X with a gentle swirling at 37 °C for 15 minutes. Medium containing FBS was added to stop the reaction of trypsin. After cells disassociated, the suspension was passed through 100 µm cell strainer. Cell suspension was centrifuged at 1500 rpm for 5 minutes and cells were counted. Keratinocytes were plated at 20,000-30,000 cells/cm^2^ in a tissue culture plate (TCP) precoated with collagen. TCP was coated with 50 µg/ml of collagen I from rat tail for an hour at room temperature. Keratinocytes were initially grown in serum free medium (KSFM) supplemented with human recombinant epidermal growth factor (EGF-1-53) and bovine pituitary extract (BPE) (Life technologies, Grand Island, NY), 1% ABAM and 10% FBS for the first 24 hours. Media was changed to KSFM supplemented with EGF, BPE and 1% ABAM thereafter. Media was changed every two days thereafter. Cells were trypsinized when reached 70-80% confluence.

***Porcine Wound Bandaging***

Animals were sedated with 0.05 mg/kg IM dexmedetomidine and maintained on isoflurane.  They were then reversed with atimpamezole (0.5 mg/kg IM). Analgesics were not necessary after bandage changes. Those animals that received biopsies were given one dose of 4 mg/kg carprofen SC. The surface and the edges of the wounds were gently cleaned with sterile gauze and saline. A small amount of triple ointment antibiotic (bacitracin zinc, neomycin and polymyxin B sulfate) was placed on sterile non-adherent sterile pads and wounds were covered. Tegaderm absorbent clear acrylic dressing of 20x20 cm (3M, St. Paul, MN) was placed on the pads to secure them in place. Coban self-adherent wrap of 150 mm X 4.5 m was wrapped around the wounds to protect the primary dressings. A layer of non-sterile tubular stockinette was as used to provide additional protection for the wounds from dirt in case the primary dressing slides. A firm plastic shell secured with soft straps was placed on the top of the dressing to prevent any damage to the wounds from rubbing. Finally, a custom made jacket was placed to hold the dressing in place. Bandages were changed twice a week for 8 weeks under aseptic procedures.

**Supplementary Figures**

**Fig. S1.** Porcine study design and time-line. (A) After harvesting 10x10cm partial thickness skin biopsies, keratinocytes and fibroblasts were isolation expanded in culture for 3-7 days. (B) Four 10x10cm full thickness excisional wounds were created per animal (n=6), and *in situ* bioprinting of layered cell constructs or control treatments was performed (C). (D) Non-invasive analysis of wound healing included imaging and measurements of wound size, contraction and re-epithelialization. Biweekly biopsies were performed for histological analysis of epidermal and dermal structure and composition.

**Fig. S2.** Inflammation grading criteria for evaluation of the acute (AI) and chronic (CI) inflammatory response of treatments. A pathologist blinded to the treatment groups scored acute and chronic inflammation in H&E stained sections using the grading criteria shown.

**Fig S3.** (A) Images of wound healing of bioprinted autologous keratinocytes and fibroblasts compared to sprayed bioprinted autologous keratinocytes and fibroblasts using a FibriJet biomaterial delivery device with a gas-assisted dual component applicator spray tip. Although bioprinted wounds appeared to have reduced contraction, increased re-epithelialization and more rapid wound closure, however there was no statistical differences for these parameters at any time-point. (B) Bioprinted wounds showed earlier epithelialization with almost complete coverage of the wound at 2 weeks, while wounds receiving cells using the spraying methodology did not show a formation of an epidermis until week 4. (C) Trichrome staining confirmed that bioprinted wounds showed accelerated formation of epidermis and more mature dermis tissue and blue stained collagen fibers were more prominent in bioprinted wounds at week 4, compared to wounds treated with sprayed cells.


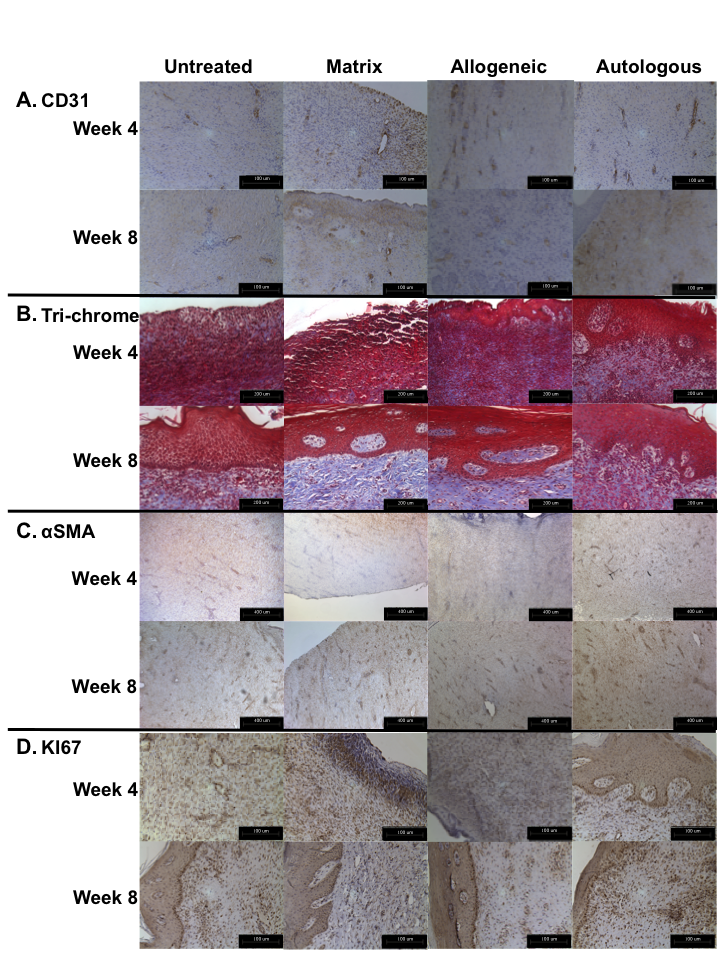


**Fig. S4.** Additional histological images for vascularization, collagen deposition, myofibroblast activation, and cell proliferation. (A) CD31-positive blood vessels. (B) Trichrome staining of blue stained collagen fibers. (C) αSMA-positive cells. (D) Ki67 positive proliferating cells. CD31/Ki67: Magnification 20x, Scale bars 100µm, Trichrome: Magnification 40x, Scale bars 200µm, αSMA: Magnification 80x, Scale bars 400µm.
